# A Systematic Review of Criminal Recidivism Rates Worldwide: Current Difficulties and Recommendations for Best Practice

**DOI:** 10.1371/journal.pone.0130390

**Published:** 2015-06-18

**Authors:** Seena Fazel, Achim Wolf

**Affiliations:** University of Oxford, Department of Psychiatry, Warneford Hospital, Warneford Lane, Headington, Oxford, United Kingdom; Universidad Veracruzana, MEXICO

## Abstract

**Objectives:**

To systematically review recidivism rates internationally, report whether they are comparable and, on the basis of this, develop best reporting guidelines for recidivism.

**Methods:**

We searched MEDLINE, Google Web, and Google Scholar search engines for recidivism rates around the world, using both non-country-specific searches as well as targeted searches for the 20 countries with the largest total prison populations worldwide.

**Results:**

We identified recidivism data for 18 countries. Of the 20 countries with the largest prison populations, only 2 reported repeat offending rates. The most commonly reported outcome was 2-year reconviction rates in prisoners. Sample selection and definitions of recidivism varied widely, and few countries were comparable.

**Conclusions:**

Recidivism data are currently not valid for international comparisons. Justice Departments should consider using the reporting guidelines developed in this paper to report their data.

## Introduction

Rates of criminal recidivism are reported to be as high as 50% in many jurisdictions, and, unlike recorded crime rates in the general population, have not declined in recent years.[1] Recidivism is a broad term that refers to relapse of criminal behaviour, which can include a range of outcomes, including rearrest, reconviction, and reimprisonment. Prisoners represent a high-risk group compared to other offenders,[2] with huge associated costs and a large contribution to overall societal criminality and violence. A number of studies have tried to identify factors that influence repeat offending rates within and between countries,[3–5] but these studies are hampered by problems with sample selection, definitions of what constitutes recidivism, and the length of follow-up.

Several differences in recording and reporting practices make between-country comparisons difficult. First, definitions of outcomes vary from rearrest to reoffending to reimprisonment. Even within those definitions, countries differ in their inclusion of misdemeanours, fines, traffic offences, and other crimes. Second, samples differ and can include offenders, prisoners, and those from other open or closed institutions. Finally, no consistent follow-up times are used and these generally vary between 6 months and 5 years.

Recidivism rates may actually differ between countries and may be secondary to many factors. This should be the subject of investigation, particularly if more comparable recidivism data becomes available. Possible explanations include the level of post-release supervision, the threshold for incarceration, the range and quality of intra-prison programmes, and investment into prison medical services, particularly those targeting drug and alcohol problems and other psychiatric disorders.[6]

In this paper, we have reviewed recidivism data worldwide and examined how definitions vary, in order to develop a comprehensive reporting checklist and best practice guidelines for presenting recidivism statistics that will allow for international comparisons. Our aims are to review recidivism rates, examine to what extent they are comparable, and present best practice in terms of reporting. Valid international comparisons are potentially important in providing a framework to examine the factors explaining differences in recidivism, and consider structural or service-related interventions that can be trialled to reduce reoffending rates.

## Methods

### Searches

**Fig 1:**
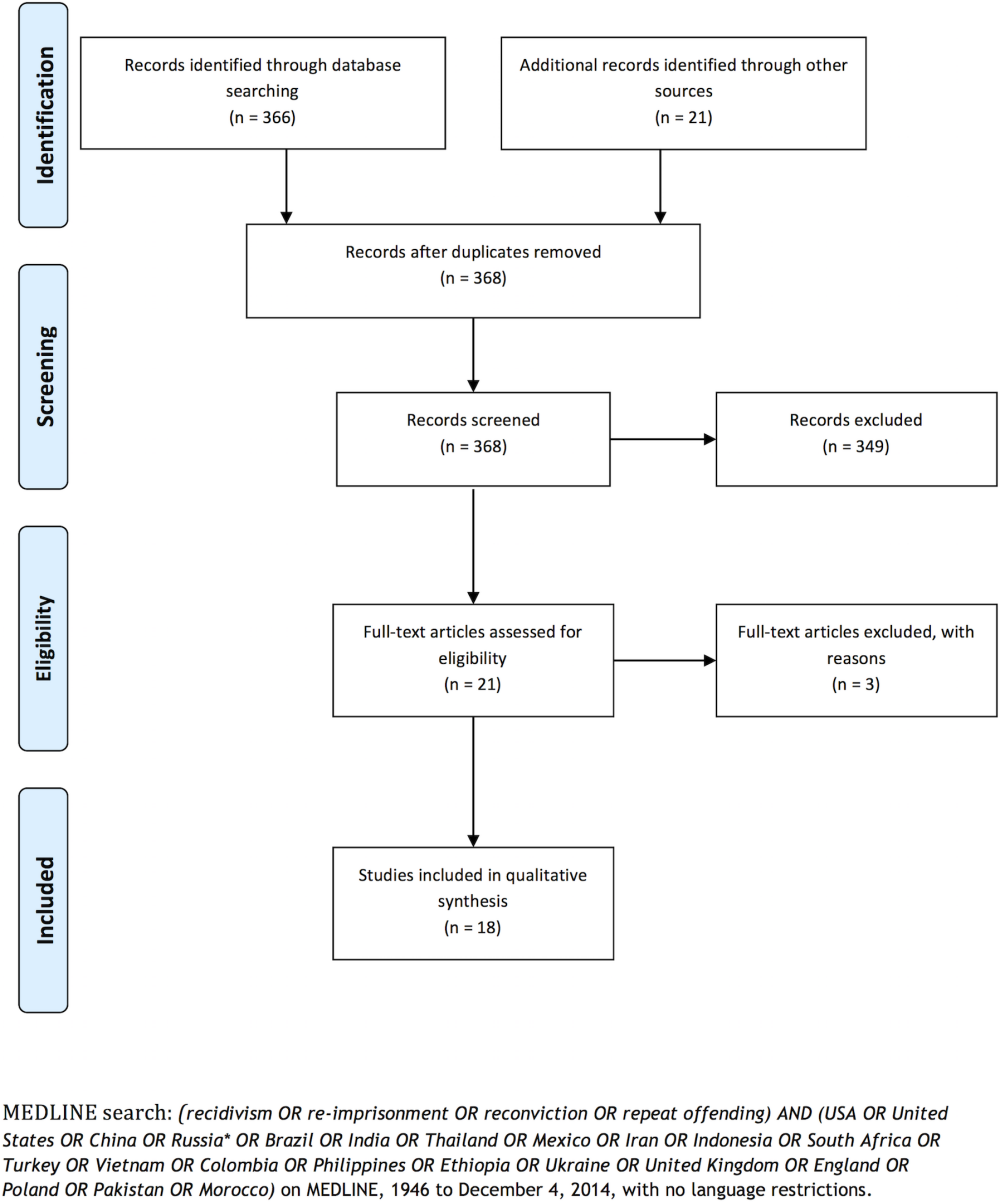
PRISMA Flow Diagram.

We searched MEDLINE, Google Web and Google Scholar search engines for recidivism rates around the world. We performed non-country-specific searches as well as targeted searches for the 20 countries with the largest total prison populations worldwide.[7] We used combinations of keywords including the country’s name, and “recidivism”, “re-imprisonment”, “reconviction”, “repeat offending”, and used no language or publication date restrictions (Fig 1). We used the most recent relevant report. Criminal justice systems were contacted for data, and clarification where necessary. Reference lists of included documents were also scanned.

### Inclusion criteria

#### Geographical

We extracted official national data that was identified through search results. Studies reporting recidivism rates for geographical regions within a country were reported when no national data were found.

#### Outcome measurements

Measurements of recidivism included in this study were rearrest, reconviction, and re-imprisonment. All time periods were reported.

#### Populations

Samples had to be solely prisoners to be included. Studies examining recidivism following suspended or other non-custodial sentences, or heterogeneous samples where rates in prison subgroups were not provided, were excluded.

#### Data extraction

Achim Wolf and Ravi Ramessur (clinical medicine student, University of Oxford) extracted the data and wrote to criminal justice systems to clarify data when necessary. We scanned titles and abstracts of publications and removed those that did not report recidivism statistics. Uncertainties were checked by Seena Fazel. We extracted data on country, rates, definitions of recidivism, and sample selection.

### Subgroup comparison

In a further table, we compared definitions of recidivism for the most reported follow-up time (2 years).

PRISMA guidelines were followed (S1 Table).

## Results

Our searches returned recidivism statistics for 21 countries. No additional regional statistics were found. Three countries were excluded due to unclear reporting on follow-up length and how recidivism was defined.[8–10] Follow-up periods varied from 6 months to 9 years. Recidivism rates were reported as reconviction (Table 1) and reimprisonment (Table 2). Reconviction data was limited to high-income countries. For re-imprisonment, information on Chile, Israel and South Korea was available in addition to European, North American and Australasian countries. The most commonly reported statistics were 2-year reconviction rates.

**Table 1:** Reconviction rates.

Country	Selection Period	Sample	Period	Rate
Canada [16]	1994–95	Prisoners	2 years	41%
Denmark [12]	2005	Prisoners	2 years	29%
Finland [12]	2005	Prisoners	2 years	36%
France [17]	2002	Prisoners	5 years	59%
Germany [18]	2004	Prisoners	3 years	48%
Iceland [12]	2005	Prisoners	2 years	27%
Ireland [19]	2013	Prisoners	3 years	51%
Netherlands [20]	2007	Prisoners	2 years	48%
Norway [12]	2005	Prisoners	2 years	20%
Singapore [21]	2011	Prisoners	2 years	27%
Sweden [12]	2005	Prisoners	2 years	43%
US [22]	2005–2010	Prisoners	6 months	13%
	2005–2010	Prisoners	1 year	23%
	2005–2010	Prisoners	2 years	36%
	2005–2010	Prisoners	3 years	45%
	2005–2010	Prisoners	4 years	51%
	2005–2010	Prisoners	5 years	55%
UK – England/Wales	2000 [1]	Prisoners	1 year	46%
	2000 [1]	Prisoners	2 years	59%
	2000 [1]	Prisoners	3 years	66%
	2000 [1]	Prisoners	4 years	70%
	2000 [1]	Prisoners	5 years	72%
	2000 [1]	Prisoners	6 years	74%
	2000 [1]	Prisoners	7 years	76%
	2000 [1]	Prisoners	8 years	77%
	2000 [1]	Prisoners	9 years	78%
	2013 [23]	Prisoners	1 year	45%
UK – Scotland [24]	2009–10	Prisoners	1 year	46%
UK – Northern Ireland [25]	2005	Prisoners	6 months	9%
	2005	Prisoners	1 year	25%
	2005	Prisoners	2 years	47%

**Table 2:** Reimprisonment rates.

Country	Selection Period	Sample	Period	Rate
Australia [26]	2009–10	Prisoners	2 years	39%
Chile [27]	2007	Prisoners	3 years	50%
France [17]	2002	Prisoners	5 years	46%
Germany [3]	2004	Prisoners	3 years	35%
Israel [4]	2004	Prisoners	5 years	43%
New Zealand [28]	2002–03	Prisoners	6 months	18%
	2002–03	Prisoners	1 year	26%
	2002–03	Prisoners	2 years	37%
	2002–03	Prisoners	3 years	44%
	2002–03	Prisoners	4 years	49%
	2002–03	Prisoners	5 years	52%
South Korea [29]	2002	Prisoners	3 years	24%
US [22]	2005–2010	Prisoners	6 months	10%
	2005–2010	Prisoners	1 year	17%
	2005–2010	Prisoners	2 years	29%
	2005–2010	Prisoners	3 years	36%
	2005–2010	Prisoners	4 years	41%
	2005–2010	Prisoners	5 years	45%

Reporting definitions varied widely, and were often not transparent (for 2-year reconviction rates, see Table 3).

**Table 3:** Offence types included and excluded in reported 2-year reconviction rates.

Country	Rate	Includes	Excludes	Uncertainties	Incarceration rates [7]*
**Canada**	41%	Offences resulting in fines or provincial sentences			118
**England and Wales**	59%	Fines			148
**Netherlands**	48%	Cases not yet settled and on appeal. Fines	Minor offences		82
**Northern Ireland**	47%	Fines	‘Pseudo-reconvictions’		101
**Singapore**	27%			Fines, minor offences, traffic offences	230
**USA**	36%	Jails as outcome	Jails as index disposal	Fines	716
			Traffic offences		
**Nordic countries**					
**Denmark**	29%		Fines and misdemeanours sanctioned outside courts		73
**Finland**	36%		As above		58
**Iceland**	27%		As above		47
**Norway**	20%		As above		72
**Sweden**	43%		As above		67

* per 100,000 national population Notes: Pseudo-reconvictions are convictions which occur after the date of the index conviction but which relate to offence(s) committed prior to that date.

To address differences in definitions and measurements, we have developed reporting guidelines covering relevant aspects of repeat offending including inclusion and exclusion criteria, follow-up time, definition of recidivism, and other minimum information to allow international comparisons to be made (S2 Table). We followed principles previously used in the development of medical checklists, including a review of the literature, ease of use, and a plan for future changes to the guidelines.[11] We suggest reporting data separately by age and gender as these are factors linked to recidivism, and currently routinely collected data in many countries. Reporting should focus on adults, as the age of criminal responsibility varies.

## Discussion

Our systematic review of recidivism rates internationally has two main findings. First, few of the countries with the largest prison populations reported recidivism statistics. For example, of the 20 countries with the largest prison populations in 2010–2011[7] (i.e. the countries where successful interventions could have the greatest population impact), only two (USA and England/Wales) reported recidivism statistics, with the remaining 16 rates from other countries. By way of comparison, we included Nordic countries which have a reputation for high quality national crime statistics and low recidivism. Second, there was significant variation between countries in how recidivism was defined and reported. For example, in Norway, 2-year recidivism rates ranged from 14% to 42% depending on whether the sample included arrested, convicted or imprisoned persons and/or the outcome was arrest, conviction or imprisonment.[2] Sweden reported a 2-year reconviction rate amongst prisoners of 43%,[12] which on the surface compares favourably to 59% in England and Wales.[1] However, the latter includes fines in the reconviction measure, whereas the former does not. In a separate report including fines, the Swedish rate rises to 66%.[13] Heterogeneity in rates may be due to different definitions (especially inclusion or exclusion of fines), which may explain lower rates in some Nordic countries. However, even after accounting for this, we found no obvious relationship with incarceration rates. Any further analysis is difficult as definitions are so variable. Further work could examine possible explanations for differences across Nordic countries–where 2-year rates range from 20% (Norway) to 43% (Sweden)–and the nations of the UK (1 year rates of 25% in Northern Ireland, compared to 45% in England and Wales).

Within-country comparisons (between regions, or over time) could provide further information: a separate US report included state-specific recidivism rates for 33 states,[14] which ranged from 23% for Oregon to 61% for Minnesota. These rates appear to be relatively comparable (return to prison for a new conviction or technical violation within 3 years). A more informative comparison would include return to jails as sentencing guidelines may complicate interpretation of differences between states. For example, the average duration of imprisonment may be longer in one state compared to another. Thus, direct comparisons remain difficult due to differences in reporting of multiple releases within a year, and whether and which technical violations are included. As such, our reporting guidelines could be used for more consistent within-country comparisons.

We conclude that international comparisons are currently not valid. To allow for comparison between countries, consistency and transparency are required, and on the basis of our review, we have published a reporting checklist.

Previous reporting guidelines[15] have been published but not implemented. While they aimed to improve transparency through reporting, they did not offer recommendations of best practice and therefore did not promote comparability. Such standardization is important as accurate recidivism data would support evidence-based recidivism research, policy, and practice.

On publication of these data, we plan to compile a list of worldwide recidivism statistics. We aim to publish the first report in 2018, by collecting the relevant statistics. The checklist should be downloaded at http://www.psych.ox.ac.uk/research/forensic-psychiatry, and sent to fazel.pa@psych.ox.ac.uk. Results will be published every three years, and the checklist will be updated through feedback from justice departments and other stakeholders.

## Supporting Information

S1 Table PRISMA 2009 Checklist. (DOC)

S2 Table Recidivism Reporting Checklist. (PDF)

## References

[1] Ministry of Justice. Compendium of reoffending statistics and analysis. https://www.gov.uk/government/publications/compendium-of-reoffending-statistics-and-analysis. London: Ministry of Justice. 2012.

[2] Andersen SN, Skardhamar T. Pick a number: Mapping recidivism measures and their consequences Oslo: Statistics Norway Discussion Papers 2014.

[3] Fazel S, Yu R. Psychotic disorders and repeat offending: systematic review and meta-analysis. Schizophr Bull. 20011:37(4):800–810r. 10.1093/schbul/sbp135 PMC3122287 19959703

[4] Bonta J, Law M, Hanson K. The prediction of criminal and violent recidivism among mentally disordered offenders: a meta-analysis. Psychol Bull. 1998;123(2):123 9522681 10.1037/0033-2909.123.2.123

[5] Hanson RK, Morton-Bourgon KE. The characteristics of persistent sexual offenders: a meta-analysis of recidivism studies. J Consult Clin Psychol. 2005;73(6):1154 16392988 10.1037/0022-006X.73.6.1154

[6] McGuire J, Bilby CA, Hatcher RM, Hollin CR, Hounsome J, Palmer EJ. Evaluation of structured cognitive—behavioural treatment programmes in reducing criminal recidivism. J Exp Criminol. 2008;4(1):21–40.

[7] Walmsley R. World prison population list, 10th edition http://www.prisonstudies.org/research-publications. London: International Centre for Prison Studies; 2013.

[8] National Crime Records Bureau. Crime in India 1994. New Delhi: NCRB, 1996.

[9] Rujjanavet S. Improving the reintegration of offenders into the community: the current situation of Thai corrections. 135th International Senior Seminar Participants’ Papers. 2008; Resource Material Series No.74:142–9.

[10] Tongzhi Y. Contemporary recidivism and its control in China. 135th International Senior Seminar Participants’ Papers. 2008; Resource Material Series No.74:109–14.

[11] Hales B, Terblanche M, Fowler R, Sibbald W. Development of medical checklists for improved quality of patient care. Int J Qual Health Care. 2008;20(1):22–30. 18073269 10.1093/intqhc/mzm062

[12] Graunbøl HM, Kielstrup B, Muiluvuori M-L, Tyni S, Baldursson ES, Gudmundsdottir H, et al Retur: en nordisk undersøgelse af recidiv blant klienter i kriminalforsorgen Oslo: Kriminalomsorgens utdanningssenter; 2010.

[13] Swedish National Council for Crime Prevention. Recidivists among all persons by gender 2003–2006. http://www.bra.se/bra/bra-in-english/home/crime-and-statistics/crime-statistics/recidivismhtml. Stockholm: Swedish Council for Crime Prevention; 2012.

[14] PEW Center on the States. State of Recidivism: The Revolving Door of America’s Prisons. Washington, DC: The Pew Charitable Trusts; 2011.

[15] Wartna B, Nijssen L. Documentatiecentrum WO-e National studies on recidivism: an inventory of large-scale recidivism research in 33 European Countries. Amsterdam: Ministry of Justice, Research and Documentation Center; 2006.

[16] Bonta J, Rugge T, Dauvergne M. The Reconviction Rate of Federal Offenders 2003–02, http://www.publicsafety.gc.ca/cnt/rsrcs/pblctns/rcnvctn-rt-fdrl/index-eng.aspx. Gatineau, Quebec: Public Works and Government Services Canada 2003.

[17] Kensey A, Benaouda A. Les risques de récidive des sortants de prison Une nouvelle évaluation. Cahiers d’études pénitentiaires et criminologiques Paris: Direction de l’administration pénitentiaire; 2011 doi: 10.1107/S1600536813013548

[18] Jehle M Jr, Albrecht H Jr, Hohmann-Fricke S, Tetal C. Legalbewährung nach strafrechtlichen Sanktionen Berlin: Bundesministerium der Justiz; 2010.

[19] Central Statistics Office. Prison Recidivism: 2008 Cohort. Dublin: Central Statistics Office, Ireland; 2013.

[20] Wartna BSJ, Tollenaar N, Blom M, Alma SM, Bregman IM, Essers AAM, et al Recidivism report 2002–2008 The Hague: Dutch Ministry of Justice and Security; 2011.

[21] Singapore Prison Service. Inspire: Annual Report 2013. http://www.sps.gov.sg/sites/default/files/SPSAnnualReport10_updated_lowRes.pdf. Singapore: Singapore Prison Service; 2013.

[22] Cooper A, Durose M, Snyder H. Recidivism Of Prisoners Released In 30 States In 2005: Patterns From 2005 To 2010. http://www.bjs.gov/index.cfm?ty=pbdetail&iid=4986. Washington, DC: Bureau of Justice Statistics; 2014.

[23] Ministry of Justice. Proven reoffending statistics: April 2012 to March 2013 London: Ministry of Justice; 2015.

[24] UK Statistics Authority. Reconviction rates in Scotland: 2009–10 offender cohort http://www.scotland.gov.uk/Resource/0040/00402666.pdf. Edinburgh: UK Statistics Authority; 2012.

[25] Department of Justice. Adult Reconviction In Northern Ireland 2005 Research and Statistical Bulletin 1/2011. Belfast: Department of Justice; 2011.

[26] Australian Government Productivity Commission. Justice Sector Overview. Melbourne: Australian Governement Productivity Commission; 2013.

[27] Peillard AMM, Correa NM, Chahuán GW, Lacoa JF. La Reincidencia en el Sistema Penitenciario Chileno http://www.pazciudadana.cl/publicacion/reincidencia-en-el-sistema-penitenciario-chileno. Santiago de Chile: Fundaction Paz Ciudana; 2013

[28] Nadesu A. Reconviction patterns of released prisoners: A 60-months follow-up analysis Auckland: New Zealand Department of Corrections; 2009.

[29] Ha Y-H. Promoting public safety and controlling recidivism using effective interventions with offenders. 135th International Senior Seminar Participants’ papers. 2010: Resource Material Series No.74.

