# Supplementary material for: A Systematic Review of Criminal Recidivism Rates Worldwide: Current Difficulties and Recommendations for Best Practice
Source: PLoS One. 2015 Jun 18;10(6):e0130390. doi: 10.1371/journal.pone.0130390 (PMC4472929; doi:10.1371/journal.pone.0130390)
Supplement: S2 Table — (PDF) [file pone.0130390.s002.pdf]

S2 Table. Recidivism Reporting Checklist

COUNTRY:	
**Population:**	**Imprisoned* - Convicted - Arrested**
**Outcome:**	**Reimprisonment - Reconviction* - Rearrest**
**Follow-up:**	**1 year - 2 years* - 3 years - 4 years - 5 years**
**Rate:**	**%**

DETAILED INFORMATION

POPULATION	NOTES	RECOMMENDED	P#
**1. Inclusion criteria**			
**a. Geographical**	Are you reporting on the country as a whole, or a specific region?	Include whole country.	
**b. Index disposal**	Who is included? Imprisoned, convicted, arrested? Define offences.	Imprisoned.	
**c. Population**	What institutions? Sub-sample or whole country?	All adult prisons (age 18+). Report sample size.	
**d. Time period**	When did the observation period start?	Date of release from prison.	
**2. Exclusion criteria**			
**a. Other samples**	Who is not included? e.g. secure hospital patients, youth institutions.	Exclude individuals sent to secure hospital, prisoners under 18.	
**b. Other offences**	What is not included? e.g. misdemeanours, minor offences, antisocial behaviour.	Include all prisoners. If any exclusions, report estimated numbers.	
**c. Other disposals**	What is not included? e.g. fines, community service, other sanctions.	Include all prisoners. If any exclusions, report estimated numbers.	
**3. Sample**			
**a. Basic characteristics**	Age, gender, ethnicity, education level.	Gender. 10-year age bands (18-24, 25-34, 35-44, 45-54, 55-64, 65+).	
**b. Index offences**	What offences had been committed in the population?	Burglary/Theft, Fraud, Sexual offences (incl. rape), Violence against person, Motoring, Drugs, Other, not recorded.	
**c. Disposal**	If prisoners, what length of time did they stay?	<1 year, <5 years, <10 years, ≧10 years.	

OUTCOME	NOTES	RECOMMENDED	P#
**1. Inclusion criteria**			
**a. Geographical**	Where can the recidivism occur?	Whole country.	
**b. Outcome disposal**	Definition of recidivism? Imprisoned, convicted, arrested?	Convicted of any offence and recalls.	
**c. Follow-up**	How long was the sample followed-up for?	2 year follow-up (or more follow-up periods).	
**d. Censoring**	Was the follow-up data censored? e.g. by using survival analysis.		
**d. Date**	What date was used? e.g. date of offence, date of conviction.	Use date of offence.	
**2. Exclusion criteria**			
**a. Other offences**	What is not included? e.g. traffic offences, misdemeanours, etc.	Exclude traffic offences only.	
**b. Other disposal**	What is not included? e.g. fines, community service, suspended sentence.	Include all convictions. Exclude cautions.	

DATA	NOTES	RECOMMENDED	P#
**1. Source**			
**a. Population**	Where was the population sample data collected from?	National prison service.	
**b. Outcome**	Where was outcome data collected from?	National crime agency.	
**2. Quality**	Loss to follow-up, underreporting, misclassification etc.		
**a. Linkage**	How were offences to those released/convicted? (e.g. date of birth).	Unique identifiers.	
**b. Pseudoconvictions**	How were new convictions which related to offences before the index offence treated?	Exclude.	
**3. Funding**	Who funded the data linkage?		
